# Construction of a rice glycoside hydrolase phylogenomic database and identification of targets for biofuel research

**DOI:** 10.3389/fpls.2013.00330

**Published:** 2013-08-26

**Authors:** Rita Sharma, Peijian Cao, Ki-Hong Jung, Manoj K. Sharma, Pamela C. Ronald

**Affiliations:** ^1^Department of Plant Pathology and The Genome Center, University of California, DavisDavis, CA, USA; ^2^Feedstocks Divison, Joint BioEnergy InstituteEmeryville, CA, USA; ^3^China Tobacco Gene Research Center, Zhengzhou Tobacco Research InstituteZhengzhou, China; ^4^Department of Plant Molecular Systems Biotechnology and Crop Biotech Institute, Kyung Hee UniversityYongin, South Korea

**Keywords:** biofuel, cell wall, database, duplication, glycoside hydrolase, RiceNet, stress

## Abstract

Glycoside hydrolases (GH) catalyze the hydrolysis of glycosidic bonds in cell wall polymers and can have major effects on cell wall architecture. Taking advantage of the massive datasets available in public databases, we have constructed a rice phylogenomic database of GHs (http://ricephylogenomics.ucdavis.edu/cellwalls/gh/). This database integrates multiple data types including the structural features, orthologous relationships, mutant availability, and gene expression patterns for each GH family in a phylogenomic context. The rice genome encodes 437 GH genes classified into 34 families. Based on pairwise comparison with eight dicot and four monocot genomes, we identified 138 GH genes that are highly diverged between monocots and dicots, 57 of which have diverged further in rice as compared with four monocot genomes scanned in this study. Chromosomal localization and expression analysis suggest a role for both whole-genome and localized gene duplications in expansion and diversification of GH families in rice. We examined the meta-profiles of expression patterns of GH genes in twenty different anatomical tissues of rice. Transcripts of 51 genes exhibit tissue or developmental stage-preferential expression, whereas, seventeen other genes preferentially accumulate in actively growing tissues. When queried in RiceNet, a probabilistic functional gene network that facilitates functional gene predictions, nine out of seventeen genes form a regulatory network with the well-characterized genes involved in biosynthesis of cell wall polymers including cellulose synthase and cellulose synthase-like genes of rice. Two-thirds of the GH genes in rice are up regulated in response to biotic and abiotic stress treatments indicating a role in stress adaptation. Our analyses identify potential GH targets for cell wall modification.

## Introduction

Plant cell walls are the most abundant source of biomass and stored solar energy on Earth. Enzymatic hydrolysis of cell wall polymers into sugars with subsequent fermentation into bio-alcohols is viewed as a promising alternative to fossil fuels (Pauly and Keegstra, 2008). The identification and characterization of genes encoding enzymes that catalyze the synthesis and degradation of plant cell wall polysaccharides is needed to advance the knowledge required for production of genetically improved biofuel feedstocks (Mansfield, 2009; Lionetti et al., 2010; Yang et al., 2013).

The various enzymes involved in biosynthesis, modification and degradation of cell wall components across different taxonomic groups are catalogued in the frequently updated Carbohydrate-Active EnZymes (CAZy) database (Cantarel et al., 2009). Glycoside hydrolases (GHs), also known as glycosyl hydrolases, make up nearly half of the enzymes classified in CAZy database. GHs catalyze the hydrolysis of O- or S- glycosidic linkages to release sugars and, therefore, can make major contributions to degrading biomass for biofuel production. GHs can also affect cellulose biosynthesis and lignocellulose crystallinity modification (Xie et al., 2013); altering the expression levels of GH genes modulates cellulose levels and, therefore, biomass yield (Zuo et al., 2000; Molhoj et al., 2002; Szyjanowicz et al., 2004; Lopez-Casado et al., 2008). Although the industrial importance of GHs in deconstructing plant biomass has fueled remarkable insights into the structural and biochemical properties of representative members from different GH families in various microorganisms, our current knowledge about plant glycoside hydrolases is still in its infancy (Lopez-Casado et al., 2008). One limit to progress in this field is the fact that there are so many GH genes in plant genomes that characterizing the functions of each of them using reverse genetics approaches is not yet feasible.

Phylogenomic databases are very useful for predicting evolutionary relationships and inferring gene functions (Cao et al., 2008; Jung et al., 2010). In this study, we report construction of a phylogenomic database for rice GHs (http://ricephylogenomics.ucdavis.edu/cellwalls/gh/) and a comprehensive *in silico* analysis to identify key targets for advancing biofuel research. The GH database hosts and displays the information about annotation, structural features, orthologous relationships, mutant availability, and gene expression patterns of GH genes in rice in phylogenetic context. We utilized the inparanoid algorithm (http://inparanoid51.sbc.su.se/cgi-bin/index.cgi), developed for eukaryotic orthology analysis, to identify GH genes that are highly diverged in rice compared to other dicots and monocot species. The meta-analysis tool of Rice Oligonucleotide Array Database (ROAD; http://www.ricearray.org/), designed to allow easy extraction of gene expression profiles across 1867 publicly available microarray datasets (Cao et al., 2012), allowed us to analyze the expression patterns of all GH genes in twenty different anatomic tissues of rice. A set of seventeen GH genes exhibiting preferential expression in actively growing tissues were queried, using a recently developed web-based probabilistic functional networks tool called RiceNet (Lee et al., 2011), to infer their functional relevance in rice. The short list of genes identified herein provides a good starting point for functional studies that will increase our understanding of and ability to manipulate grass cell wall qualities.

## Results and discussion

### Classification of 437 glycoside hydrolase genes in rice into 34 families

When accessed for this study, the CAZy database (http://www.cazy.org/Glycoside-Hydrolases.html) contained 430 genes annotated as coding for glycoside hydrolases in rice. Using homology and domain searches (see Material and Methods), we identified an additional seven family members resulting in 437 GH genes in rice. One hundred and three of these encode multiple transcripts due to alternative splicing; the total number of transcripts encoded by rice GH genes is therefore, 614. We classified these genes into 34 of the pre-defined 132 GH gene families in the CAZy database (Table 1).

**Table 1:** **The classification and characteristic features of glycoside hydrolase families in rice**.

**S. no.**	**Family**	**Total number of genes**	**Number of genes showing splicing**	**Total number of transcripts**	**Fold**	**Clan**	**Mechanism**	**Conventional activities in plants**
1	GH1	37	20	74	(β/α)_8_	GH-A	Retaining	β-glucosidase, myrosinase, β-mannosidase, β-D-fucosidase, β-glucoronidase, isoflavonoid 7-O-β-apiosyl-β-glucosidase, thioglucosidase, strictosidine β-glucosidase, raucaffricine β-glucosidase, β-primeverosidase, and prunasin β-glucosidase
2	GH2	2	1	3	(β/α)_8_	GH-A	Retaining	β-mannosidase, β-galactosidase, and β-glucoronidase
3	GH3	16	5	30		Unknown	Retaining	β-xylosidase, β-glucosidase, α-L-arabinofuranosidase, β-N-acetylhexosaminidase, glucan 1,3-β-glucosidase, glucan 1,4-β-glucosidase, a-L-arabinofuranosidase, and exo-1,3-1,4-glucanase
4	GH5	17	4	21	(β/α)_8_	GH-A	Retaining	β-mannanase and β-glucosidase
5	GH9	26	3	29	(α/α)_6_	Unknown	Inverting	Endoglucanase
6	GH10	14	3	18	(β/α)_8_	GH-A	Retaining	Endoxylanase
7	GH13	17	8	33	(β/α)_8_	GH-H	Retaining	Alpha-amylase, isoamylase, pullulanas, and branching enzyme
8	GH14	11	0	11	(β/α)_8_	Unknown	Inverting	β-amylase
9	GH16	31	5	38	β-jelly roll	GH-B	Retaining	Xyloglucan endotransglycosylase hydrolase
10	GH17	68	20	103	(β/α)_8_	GH-A	Retaining	Endo-β-1,3-glucosidase, β-1,3-glucanosyltransglycosylase and licheninase
11	GH18	35	0	35	(β/α)_8_	GH-K	Retaining	Chitinase
12	GH19	20	1	21		Unknown	Inverting	Chitinase
13	GH20	6	1	8	(β/α)_8_	GH-K	Retaining	β-N-acetylhexosaminidase
14	GH27	5	2	9	(β/α)_8_	GH-D	Retaining	α-galactosidase
15	GH28	44	2	49	β-helix	GH-N	Inverting	Polygalacturonase
16	GH29	2	0	2	(β/α)_8_	Unknown	Retaining	α-L-fucosidase
17	GH31	7	1	8	(β/α)_8_	GH-D	Retaining	α-xylosidase, α-glucosidase, and isomaltosyltransferase
18	GH32	11	4	16	5-fold β-propeller	GH-J	Retaining	Invertase, fructan exohydrolase, inulinase, sucrose:sucrose 1-fructosyltransferase, and fructan β-(2,6)-fructosidase
19	GH33	3	0	3	6-fold β-propeller	GH-E	Retaining	Not determined
20	GH35	15	5	23	(β/α)_8_	GH-A	Retaining	β-galactosidase
21	GH36	10	0	10	(β/α)_8_	GH-D	Retaining	α-galactosidase, stachyose synthase, and raffinose synthase
22	GH37	1	0	1	(α/α)_6_	GH-G	Inverting	α,α-trehalase
23	GH38	3	3	8	(β/α)7	Unknown	Retaining	α-mannosidase
24	GH43	2	1	4	5-fold β-propeller	GH-F	Inverting	β-xylosidase, α-L-arabinofuranosidase, xylanase, and galactan 1,3-β-galactosidase
25	GH47	4	1	7	(α/α)_7_	Unknown	Inverting	α-mannosidase
26	GH51	8	4	17	(β/α)_8_	GH-A	Retaining	α-L-arabinofuranosidase and β-xylosidase
27	GH63	1	1	2	(α/α)_6_	GH-G	Inverting	α-glucosidase
28	GH77	2	0	2	(β/α)_8_	GH-H	Retaining	Amylomaltase
29	GH79	5	3	8	(β/α)_8_	GH-A	Retaining	β-glucoronidase
30	GH81	1	0	1		Unknown	Inverting	Endo-β-1,3-glucanase
31	GH85	2	0	2	(β/α)_8_	GH-K	Retaining	Endo-β-N-acetylglucosaminidase
32	GH89	1	0	1	(β/α)_8_	Unknown	Retaining	α-N-acetylglucosaminidase
33	GH95	2	1	3	(α/α)_6_	Unknown	Inverting	α-1,2-L-fucosidase
34	GH100	8	3	14		Unknown	unknown	Invertase

This classification was primarily based on sequence similarity as opposed to substrate specificity; therefore, most of these families are polyspecific because enzymes with different substrate specificities are grouped together (Henrissat, 1991; Henrissat and Bairoch, 1993; Henrissat and Davies, 1997). Further, families having conserved three-dimensional structure, catalytic geometry, and reaction stereochemistry have been grouped into clans (Henrissat and Bairoch, 1996). Fourteen clans named from GH-A to GH-N, have been defined for glycoside hydrolases in the CAZy database (http://www.cazy.org/Glycoside-Hydrolases.html). Out of the 34 families of glycoside hydrolases in rice, 23 were categorized into 10 of the known clans, while the remaining 11 do not fall into any of the existing clans (Table 1). The most commonly occurring structural fold in glycoside hydrolases in rice is the (β/α)_8_ barrel, characteristic of 17 GH families belonging to the GH-A, GH-D, GH-H or GH-K clans as well as of the GH14, GH29 and GH89 families (http://www.cazy.org/Glycoside-Hydrolases.html). Other folds observed in glycoside hydrolases include the (α/α)_6_ in GH9, GH37, GH63 and GH95, the β-jelly roll in GH16, the 6-fold β-propeller in GH33, the 5-fold β-propeller in GH32 and GH43, the β-helix fold in GH28, the (β/α)_7_ in GH38 and the (α/α)_7_ in the GH47 family (http://www.cazy.org/Glycoside-Hydrolases.html).

Classification based on the stereochemical outcome of hydrolysis had been previously proposed for glycoside hydrolases (Koshland, 1953). The families in GH clans A, B, D, E, H, J and K act through a retaining mechanism wherein the anomeric configuration is retained in the product, whereas the mechanism of action of GH clans F, G, and N is characterized by inversion of the product's anomeric configuration. Table 1 lists the stereochemical mechanism of action as well as the number of genes, transcripts, structural folds, and known enzymatic activities for all 34 families of rice glycoside hydrolases.

### Construction of a phylogenomic database of rice glycoside hydrolases

**Figure 1:**
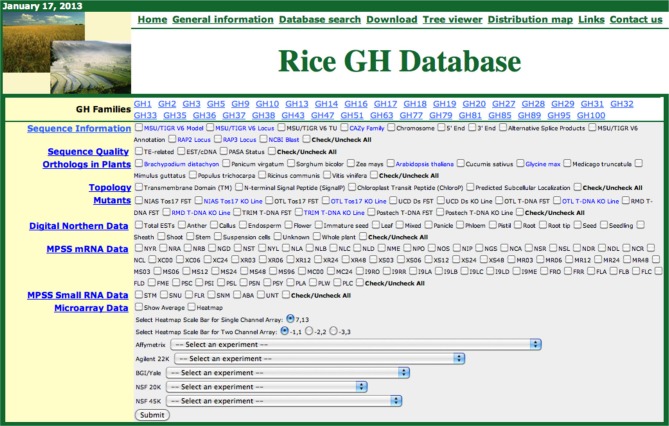
**Screen shot of the rice glycoside hydrolase (GH) database.** The functional genomic data for each GH family can be retrieved, in phylogenomic context, by selecting the gene family, checking the relevant boxes and clicking the submit button.

High-throughput characterization of cell-wall-related genes in plants is hindered due to the large numbers of genes involved and the presumed genetic redundancy in their functions. With the rapid advancements in analytical and functional genomics tools, large amounts of sequence and transcriptome information are accumulating in online public databases that can be utilized to prioritize candidate genes, predict functional redundancy, and infer gene functions. However, all these data are scattered across multiple databases therefore, requiring significant effort for integration and analysis. To assist functional genomic studies of cell wall- and stress-response-related genes in the monocot model system rice, we previously constructed phylogenomic databases for rice kinases (Dardick et al., 2007; Jung et al., 2010) and glycosyltransferases (Cao et al., 2008). We have now constructed a similar platform for rice glycoside hydrolases. Figure 1 presents a screenshot of the Rice GH database.

The Rice GH database can be accessed from a webpage shared with our other rice phylogenomic databases: http://ricephylogenomics.ucdavis.edu/. Links to general information, database descriptions, and the databases themselves are provided on the homepage of GH database (http://ricephylogenomics.ucdavis.edu/cellwalls/gh/). For each GH gene family, comprised of more than three members, a maximum likelihood phylogenetic tree has been generated that includes all the gene models (alternative splice forms). These trees can be viewed by simply clicking on the name of the respective gene family. The phylogenetic tree viewer link from GH database allows users to select a particular gene family and retrieve the desired information for all the members of that gene family by selecting relevant options (Figure 1). The data, in a phylogenetic context, are displayed in a spreadsheet format and can be easily transferred to other applications for further analysis.

The data in the Rice GH database has been integrated in a user-friendly manner. Information about Michigan State University's Rice Genome Annotation Project (RGAP; http://rice.plantbiology.msu.edu/) and, the Rice Annotation Project Database (RAP-DB; http://rapdb.dna.affrc.go.jp/) locus IDs and annotations, chromosomal position (chromosome number and 5′/3′ attributes) and alternative splice products, have been integrated and can be accessed using the “Sequence Information” tab. The locus IDs comprise hyperlinks to detailed information pages associated with the corresponding databases. The names of the gene families are hyperlinked to the descriptions of the respective families in the CAZy database. In addition, for each gene a hyperlink to the National Center for Biotechnology Information (NCBI) BLAST site (http://blast.ncbi.nlm.nih.gov/) provides users with the results of BlastP searches of that particular gene in NCBI. Information about matching transposable elements and EST/cDNAs and the Program to Assemble Spliced Alignments (PASA) (http://pasa.sourceforge.net/) status of all the genes can be accessed using the “Sequence Quality” tab. PASA exploits alignments of expressed transcripts to model gene structures and maintain gene structure annotations consistent with currently available experimental data. Information about orthologs of rice genes in eight dicot and four monocot genomes is integrated and can be accessed using the “Orthologs in Plants” tab. The “Topology” tab provides the information regarding predicted structural features of the proteins including transmembrane domains, N-terminal signal peptides, chloroplast peptides, and predicted subcellular localization. Information about the mutants available for the rice genes is integrated under the “Mutants” tab. We have also integrated diverse types of data from expression analyses including digital northern data, Massively Parallel Signature Sequencing (MPSS) mRNA data, MPSS small RNA data, and microarray data from five different platforms including Affymetrix, Agilent 22K, BGI/YALE, NSF20K, and NSF45K. Microarray-based expression data can be downloaded in spreadsheet format or viewed as heat maps by selecting the dataset from a drop-down menu (http://ricephylogenomics.ucdavis.edu/cellwalls/gh/tree.php). The integration of all these data in the Rice GH database provides a valuable resource for the rice community for conducting detailed investigations of GH genes in rice and would also guide future research directions in potential biofuel crops.

### Sequence divergence of glycoside hydrolase genes in rice

In line with the extensive diversity in structures and functions of carbohydrates, the genes encoding enzymes regulating carbohydrate metabolism are expected to have undergone significant divergence in different plant species. The glycoside hydrolases comprise a significant portion of the cell wall metabolism machinery in plants (McCann and Rose, 2010); there are 437 genes in rice, 390 in *Arabidopsis*, 356 in *Brachypodium* and 404 genes in sorghum that code for GH enzymes (Tyler et al., 2010). GH genes from all four of these genomes fall into the same 34 families as in rice (see above) but the number of genes in each family varies greatly among different plant species (Yokoyama and Nishitani, 2004; Tyler et al., 2010). Tyler et al. (2010) reported differences as much as 4-folds in the number of genes in the GH1, GH5, GH13, GH18, GH19, GH28 and GH51 families between dicot and monocot genomes. For example, the GH1 and GH28 families, encoding glucosidases, and polygalacturonases, respectively, have expanded substantially in *Arabidopsis* relative to monocot genomes, whereas, there are far more genes encoding alpha amylases (GH13), chitinases (GH18) and arabinofuranosidases (GH51) in monocot genomes. Most of these differences have been attributed to compositional differences in the cell walls of dicots and monocots (Yokoyama and Nishitani, 2004). The primary cell walls in dicots and commelinoid monocots, classified as type I, comprise primarily of the cellulose and xyloglucans embedded in pectin matrix (Carpita and Gibeaut, 1993). The major hemicellulose in the type II cell walls of commelinoid monocots including rice, on the other hand, is glucuronoarabinoxylans and these walls also contain high amounts of cellulose but negligible amounts of pectins (Carpita, 1996).

**Figure 2:**
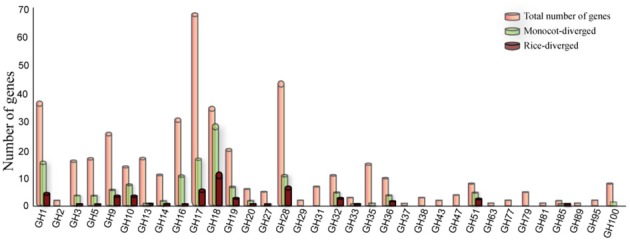
**Distribution of monocot- and rice–diverged glycoside hydrolase genes in rice.** The GH gene families are indicated on the X-axis and the Y-axis represents the number of genes. The number of rice genes diverged from eight dicot (green bars) and four monocot genomes scanned in this study (maroon bars) are shown relative to total number of genes (orange bars) in each family.

Comparison of the sizes of gene families in different genomes can indicate lineage-specific expansion or loss but not the functional divergence of corresponding family members. In this study, we used the pairwise sequence similarity scoring system (O'brien et al., 2005) to infer the orthology relationships between rice GHs and those in eight dicot (*Arabidopsis thaliana, Cucumis sativus, Glycine max, Medicago truncatula, Mimulus guttatus, Populus trichocarpa, Ricinus communis, Vitis vinifera*) and four monocot (*Brachypodium distachyon, Panicum virgatum, Sorghum bicolor, Zea mays*) genomes. We did not identify a corresponding ortholog for 138 of the 437 GH genes in rice in any of the eight dicot genomes analyzed (Table S1); we assume, therefore, that these genes have diverged to cater to monocot-specific functions. Especially in the GH1, GH10, GH18, GH32, and GH51 families, substantial numbers of the gene family members fell into the monocot-diverged category (Figure 2). Further, 57 of these 138 genes did not have an ortholog in any of the four other monocot genomes analyzed and, therefore, have diverged within the rice genome (Figure 2). Since the composition of hemicellulose varies substantially between dicot and monocot cell walls, it is not surprising that the gene families for example, GH10, GH16, GH32, and GH51 that encode enzymes to hydrolyze hemicellulose are among those with the greatest numbers of monocot-diverged genes. Chitinases, encoded by members of the GH18 family, which is comprised primarily of the monocot-diverged genes (Figure 2), are implicated in plant-pathogen interactions (Sasaki et al., 2006) and may have diversified to respond to a wide variety of pathogens.

Monocot and dicot genomes have accumulated many other molecular-level differences likely related to their different morphology, biochemistry, development and adaptation to environmental stresses during the ~200 million years since their divergence from a common ancestor (Paterson et al., 2004). The monocot- and rice-diverged GH genes identified in this study provide good candidates for understanding the differences in plant cell wall architectures and, therefore, for manipulating the qualities of grass cell walls. The ability to closely examine the expression profiles of these candidate genes to reveal, for example, preferential expression of some of them in seedlings, roots, anthers, palea/lemma or seed developmental stages (Figure S1), will also contribute to our basic understanding of plant cell wall dynamics.

### Segmental and tandem duplications played a significant role in the expansion and diversification of glycoside hydrolase genes in rice

The GH genes in rice comprise 34 families (Table 1). However, the numbers of genes in different families varies considerably; GH17, the largest family, has 68 members, whereas, GH37, GH63, GH81 and GH89 comprise only one gene each (Table 1). As evident from the analysis of orthologous sequences (Figure 2), diversity in numbers of monocot-diverged and rice-diverged genes is also apparent among the 34 GH gene families.

**Figure 3:**
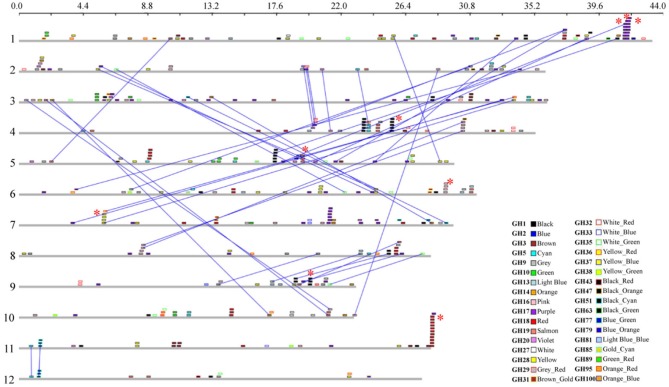
**Chromosomal localization and gene duplication of glycoside hydrolase genes in rice.** The scale at the top, in Mb, is to show the relative lengths of the rice chromosomes. Chromosome numbers are indicated on the left. Each GH family is represented by a different color; the color code key is given in the lower right side of the figure. Dashed lines connect paralogous genes on duplicated segments of the genome. The red asterisks indicate tandemly duplicated gene clusters.

**Figure 4:**
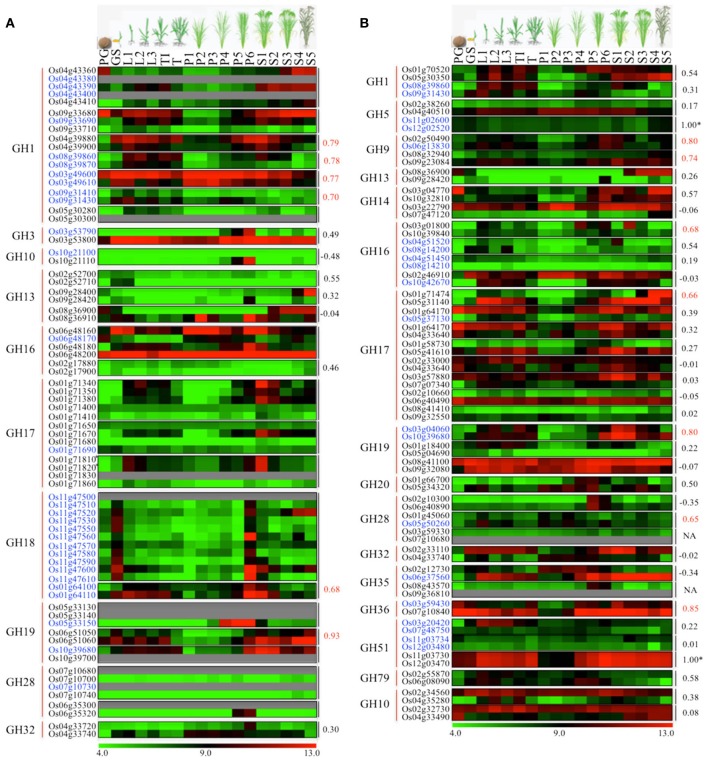
**Comparison of expression profiles of duplicated glycoside hydrolase genes in rice.** Heat maps showing expression patterns of **(A)** tandem and **(B)** segmental duplicated GH genes. The top row represents developmental stages as follows: PG, pre-germination seed; GS, germinating seedling; L1, 1st leaf; L2, 2nd leaf; L3, 3rd leaf; TI, Tillering initiation; T, Tillering stage; P1-P6, temporal stages of panicle development and S1-S5, temporal stages of seed development. The tandem/segmental duplicated gene clusters are marked by gray lines on the right. The gene families (marked by red lines) and locus IDs are listed on the left. The Pearson's correlation coefficient values among expression patterns of duplicated gene pairs are given on the right side of the heat maps wherever applicable; red indicates significant correlation. The ^*^marks duplicated genes which were represented by the same probe set ID on the Affymetrix arrays due to high sequence similarity. Locus IDs of monocot-diverged genes are given in blue. The color legend is given at the base where green signifies very low-level expression, black indicates medium-level expression, and red represents high-level expression. The numbers in the color legend correspond to relative log_2_ expression values.

One of the key mechanisms of gene expansion and diversification in plant genomes is duplication. It has been suggested that the rice genome has undergone whole-genome duplication, a more recent segmental duplication between chromosomes 11 and 12 and numerous small-scale duplications (Yu et al., 2005; Throude et al., 2009). To check the impact of these gene duplications on expansion and diversification of glycoside hydrolases in rice, we localized all the GH genes in rice (Figure 3). Genes tandemly arrayed on rice chromosomes were further analyzed for the percent homology shared among their protein sequences. Based on our criteria (see Materials and methods), we identified nine clusters of GH genes, each comprising three to eleven genes (Figure 3). The largest cluster is on chromosome 11 and comprises eleven genes encoding chitinases (GH18), whereas, three clusters of GH17 family genes were located on chromosome 1 (Figure 3). Although the eleven GH18 family genes comprising the largest cluster exhibit similar expression patterns, most of the genes from the other gene clusters, identified in this study, have diverged in their expression patterns (Figure 4A). The additional sixteen pairs of GH genes occurring in tandem and having more than 60% identity in their protein sequences (Table S2) also likely originated from tandem duplications. Of the sixteen pairs of these tandemly duplicated genes, both the partners of thirteen of them were represented on the Affymetrix array. Six of these thirteen gene pairs exhibit very similar expression profiles with a Pearson's correlation coefficient of 0.68 or more (Figure 4A).

In addition, 38 pairs of GH genes were located on segmental duplicated regions of rice genome. Rice expression data for 36 of these are available on Affymetrix arrays. Seven pairs of these genes exhibit very similar expression patterns, with correlation coefficients of 0.65 or more, whereas the other 26 pairs seem to have undergone significant divergence in their expression profiles and hence functions (Figure 4B). Eight pairs of segmentally duplicated genes belong to the GH17 family, four to GH16, and three each to GH19, GH28 and GH51 gene families.

We then compared the monocot-diverged and rice-diverged genes with the duplicated gene sets. This analysis revealed that 35% of the monocot-diverged and 30% of the rice-diverged genes overlapped with the duplicated gene sets (Figure 4, Tables S2, S3). The data further suggested that the segmental duplications are primarily responsible for expansion and functional divergence in the GH16 and GH51 gene families in monocots, whereas, the monocot-diverged genes in the GH1 and GH18 families have mainly resulted from localized tandem duplications (Figure 4, Tables S2, S3).

### Developmental expression patterns of rice glycoside hydrolase genes and associated pathways

Because plant growth and development is accompanied by significant reorganization of cell wall polysaccharides involving degradation of polysaccharide components as well as synthesis and integration of new polysaccharides, GH activities are implicated in various developmental processes (Minic and Jouanin, 2006). To explore the diverse functions of rice GHs during plant growth and development, we analyzed the expression meta-profiles of all the GH genes in 20 tissue types using the meta-analysis tool of ROAD (Cao et al., 2012). A total of 396 GH genes of rice are represented on the Affymetrix expression arrays and were analyzed for tissue-preferential/specific expression.

The majority of GH family genes exhibit very high-level expression with differential accumulation of transcripts in different tissue types. This result conforms to the role of glycoside hydrolases in dealing with constant modification and remodeling of cell walls in specific tissue types. Based on K-means clustering (Tavazoie et al., 1999) and some manual curation, we identified 51 genes from different GH families that exhibit preferential expression in vegetative or/and reproductive tissues. Based on their expression profiles, we categorized these 51 genes into the following five groups.

#### Group A: GH genes preferentially expressed in germinating seedlings and embryos

**Figure 5:**
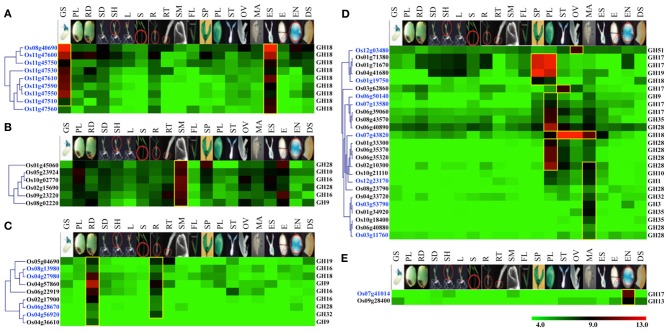
**Vegetative- and reproductive-stage-preferential expression of glycoside hydrolase genes in rice.** Five expression clusters **(A–E)** were identified based on the tissue-preferential accumulation. The anatomical tissues are given on the top as follows: GS, germinating seedling; PL, plumule; RA, radicle; SD, seedling; SH, shoot; L, leaf; S, stem; R, root; RT, root tip; SAM, shoot apical meristem; FL, flag leaf, SP, spikelet; PL, palea/lemma; ST, stigma; OV, ovary; MA, mature anther; ES, embryo sac; E, embryo; EN, endosperm; DS, dry seed. Locus IDs of monocot-diverged genes are given in blue. The color legend is given at the base; green signifies very low-level expression, black indicates medium-level expression and red represents high-level expression. The numbers in the color legend correspond to relative log_2_ expression values.

Nine chitinase-encoding genes of GH18 family, including eight from the duplicated gene cluster on chromosome 11 and one gene on chromosome 8, were categorized into group A. The transcripts of these genes preferentially accumulate in germinating seedlings and embryo sacs (Figure 5A). Moreover, all of these genes also fall in the category of monocot-diverged genes. Since, chitinases have been implicated in plant defense responses (Collinge et al., 1993), specific expression of these chitinase genes in seedlings and embryos might be part of a strategy to protect rice seedlings and embryos from fungal pathogens.

#### Group B: GH genes preferentially expressed in shoot apical meristems

Transcripts of six genes, including two encoding polygalacturonases of the GH28 family and two encoding xyloglucan endotransglycosylase/hydrolases (XTHs) of the GH16 family, primarily accumulate in shoot apical meristems (Figure 5B). Querying these genes in RiceNet (http://www.functionalnet.org/ricenet/) revealed that one of the GH28 family genes, *Os02g15690*, is expressed with the same pattern as of Class I TCP family transcription factor genes, *OsTCP4* and *OsTCP10*. The Class I TCP family genes are involved in development of axillary meristems by regulating cell division, expansion and differentiation (Martin-Trillo and Cubas, 2010). A GH9 family gene in this group, *Os08g02220*, is expressed with the same pattern as a rice *TSO1*-like gene. *Arabidopsis TSO1* is required for cytokinesis and cell expansion in reproductive tissues (Andersen et al., 2007). Since polygalacturonases and XTHs are also known to have a role in cell expansion (Rose et al., 2002; Minic, 2008), these genes are likely involved in regulating cell expansion in meristems.

#### Group C: root- and or radicle-preferentially expressed GH genes

Transcripts of nine genes primarily accumulate in radicles and mature roots (Figure 5C). These genes include two endoglucanases of the GH9 family and three xyloglucan endotransglycosylase/hydrolases (XTHs) of the GH16 family. Previous studies have implicated XTHs in root hair initiation and strengthening of cell walls in root differentiation zones (Vissenberg et al., 2001; Maris et al., 2009). Two of the genes in this category, *Os04g27980* and *Os06g28670*, are also rice-diverged and would be interesting candidates for further investigating their role(s) in root growth and development in rice.

#### Group D and E: GH genes preferentially expressed in reproductive organs

Transcripts from a set of 25 genes comprising group D preferentially accumulate in reproductive organs, with the majority of them expressed specifically in palea/lemma and/or mature anthers (Figure 5D). These include five genes encoding endo-β-1, 3-glucosidases of the GH17 family, eight polygalacturonases-encoding genes of the GH28 family and three β-galactosidase-encoding genes of the GH35 family. Polygalacturonases as well as some members of the GH35 family play a role in cell wall loosening and modification involving pectin degradation (Minic, 2008). RiceNet (http://www.functionalnet.org/ricenet/) predicted the association of the members of the GH17 family genes in this group with callose synthases of the GT48 family. Since callose deposition and degradation, as well as pectin degradation, are crucial for microsporogenesis, anthesis and pollen germination and tube growth (Taylor and Hepler, 1997; Ma, 2005), significant expression of these genes in anthers suggests their involvement in microsporogenesis and the development of the male gametophyte. Transcripts of a rice-diverged chitinase-encoding gene of the GH18 family, *Os07g43820*, accumulate in male as well as female reproductive organs. Transcripts of *Os03g62860*, a member of the GH17 family, accumulate mainly in stigmas and this gene is therefore, likely to be involved in pollen tube growth. Transcripts of a rice-diverged gene of GH51 family, *Os12g03480*, specifically accumulate in ovules. Since anthesis involves cell separation, the hydrolytic enzymes β-1,4-glucanases, α-L-arabinofuranosidases and polygalacturonases, the transcripts for which accumulate preferentially in pale/lemmas, could be involved in cell separation during anthesis (Minic, 2008).

A GH13 family member encoding alpha amylase and a monocot-diverged GH17 family member encoding β-glucanase, categorized in group E, preferentially express in rice endosperm (Figure 5E). Starch is an important carbon reserve. The branching enzymes encoded by GH13 family members have been implicated as being involved in starch synthesis as well as hydrolyzation of the starch stored in endosperm into metabolizable sugars, whereas β-glucanase is more likely to be involved in degradation of monocot-specific mixed linkage β-D-glucans in rice endosperm (Minic, 2008).

### Rice glycoside hydrolases as targets for biofuel research

In addition to their primary role in polysaccharide degradation, mounting genetic evidence suggests that several plant glycoside hydrolases are involved in biosynthesis, assembly and reorganization of cell wall polysaccharides (Minic, 2008; Sharma et al., 2011). The GH9 family genes encoding endo-1,4-β-glucanases, for example, are involved in cellulose biosynthesis (Nicol et al., 1998; Molhoj et al., 2002; Xie et al., 2013) and the xyloglucan endotransglucosylase/hydrolases (XETs) of the GH16 family have been implicated in synthesis and remodeling of xyloglucans (Rose et al., 2002; Jan et al., 2004; Yokoyama et al., 2004).

**Figure 6:**
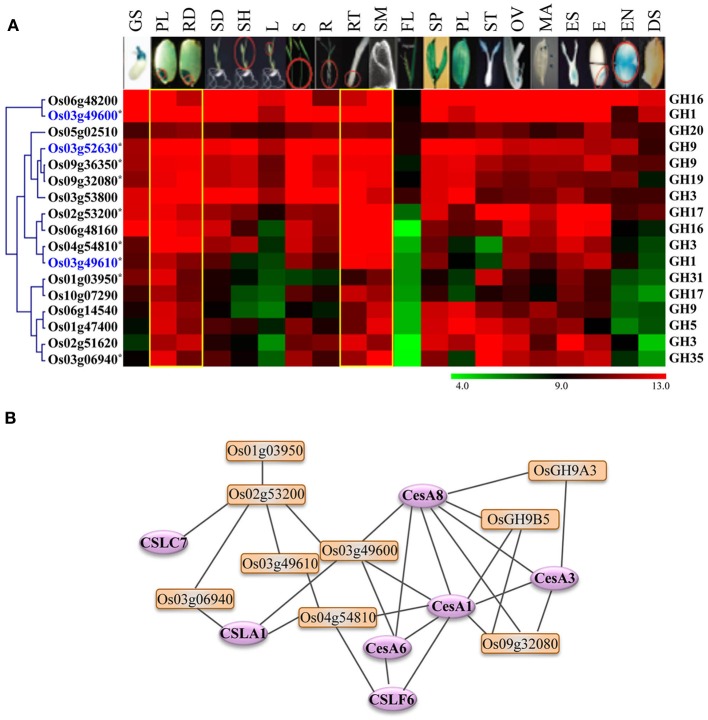
**Expression profiles and co-expression network of GH genes selected as potential targets for biofuel research. (A)** Hierarchical cluster display of expression patterns of GH genes exhibiting preferential accumulation in actively growing tissues. The anatomical tissues are given on the top as follows: GS, germinating seedling; PL, plumule; RA, radicle; SD, seedling; SH, shoot; L, leaf; S, stem; R, root; RT, root tip; SAM, shoot apical meristem; FL, flag leaf; SP, spikelet; PL, palea/lemma; ST, stigma; OV, ovary; MA, mature anther; ES, embryo sac; E, embryo; EN, endosperm; DS, dry seed. The color legend is given at the base where green signifies very low-level expression, black indicates medium-level expression, and red represents high-level expression. The numbers in the color legend correspond to relative log_2_ expression values. The locus IDs are given on the left and gene family names on the right. Locus IDs of monocot-diverged genes are given in blue. **(B)** RiceNet-predicted association among the GH genes marked by asterisk in panel **(A)** of this figure (orange boxes) and with the cellulose synthase and cellulose synthase-like genes (purple ellipses) of rice.

Because the glycoside hydrolases involved in cell wall biosynthesis are potential targets for engineering improved feedstocks for biofuel production, we took advantage of publically available metadata to identify the glycoside hydrolase genes in rice likely to have a role in cell wall biosynthesis. As genes exhibiting differential expression in vegetative and reproductive organs are likely to be dispensable for normal growth and development, we focused our analysis on genes exhibiting high-level expression in most of the tissues but preferential accumulation in tissues undergoing active cell divisions. We identified seventeen genes encoding enzymes implicated in cellulose or hemicellulose metabolism that were expressed primarily in plumule as well as radicle of seedlings, root tips and shoot apical meristems (Figure 6A). Transcripts of most of these also accumulate significantly in rice stems (Figure 6A). We queried these genes in RiceNet to determine the likelihood of their being involved in cell wall biosynthesis. Our results, based on co-expression data in rice, indicated that nine of these genes form a highly connected network with each other as well as with cellulose synthase (CesA) and cellulose synthase-like (Csl) genes of rice (Figure 6B). The major role of cellulose synthase genes is in the biosynthesis of cellulose which comprises the major component of plant cell walls (Wang et al., 2010), whereas, cellulose synthase-like genes are known to play a role in the synthesis of non-cellulose polysaccharides; CslA family members are involved in the synthesis of mannans, CslC genes in the synthesis of backbone xyloglucans and CslF genes in biosynthesis of mixed-linkage β-glucans (MLG) (Wang et al., 2010). In fact, the *CslF6* gene identified in this predictive network is the key player in synthesis of MLG in rice (Vega-Sanchez et al., 2012). The *cslf6* rice mutant displays drastic reduction in MLG content in vegetative tissues of rice and a lesion-mimic phenotype in leaves (Vega-Sanchez et al., 2012). Four genes, *Os03g52630* (*OsGH9A3*) and *Os09g36350* (*OsGH9B5*) of the GH9 family, *Os03g49600* of the GH1 family, and *Os09g32080* of the GH19 family, are highly connected to *CesA1* (*Os05g08370*), *CesA3* (*Os07g24190*), and *CesA8* (*Os07g10770*) of rice. The *Arabidopsis* orthologs of these genes are involved in primary wall cellulose biosynthesis (Persson et al., 2007). Recently, Xie et al. (2013) provided experimental evidence for role of, two of the genes shortlisted in this analysis, *OsGH9A3* and *OsGH9B5* in cellulose biosynthesis and thereby, support the results of our predictive analysis. *OsGH9A3* is also one of the rice-diverged genes identified in this study.

The genes we have identified provide a short list of strong candidates worthy of further evaluation. The roles these genes play in cell wall biosynthesis could be exploited to increase the cellulose content of grass cell walls. For example, it has been suggested that some of the GH9 family members regulate the extent to which cellulose is crystalized (Takahashi et al., 2009). Therefore, altering the expression patterns of these genes could shift the ratio of the less soluble crystalline form of cellulose to the more soluble amorphous form and lead to significant increase in the efficiency of saccharification (Abramson et al., 2010).

### Role of rice glycoside hydrolases in stress adaptation

Because the cell wall is the plant's primary defense against diverse environmental stresses and pathogens, modification of cell wall proteins is an important aspect of plant acclimation to environmental stresses (Sasidharan et al., 2011). Structural changes in cell walls mediated by cell-wall-modifying proteins help plants survive under adverse environmental conditions by regulating plant growth, providing a physical barrier or releasing products that trigger defense signaling.

**Figure 7:**
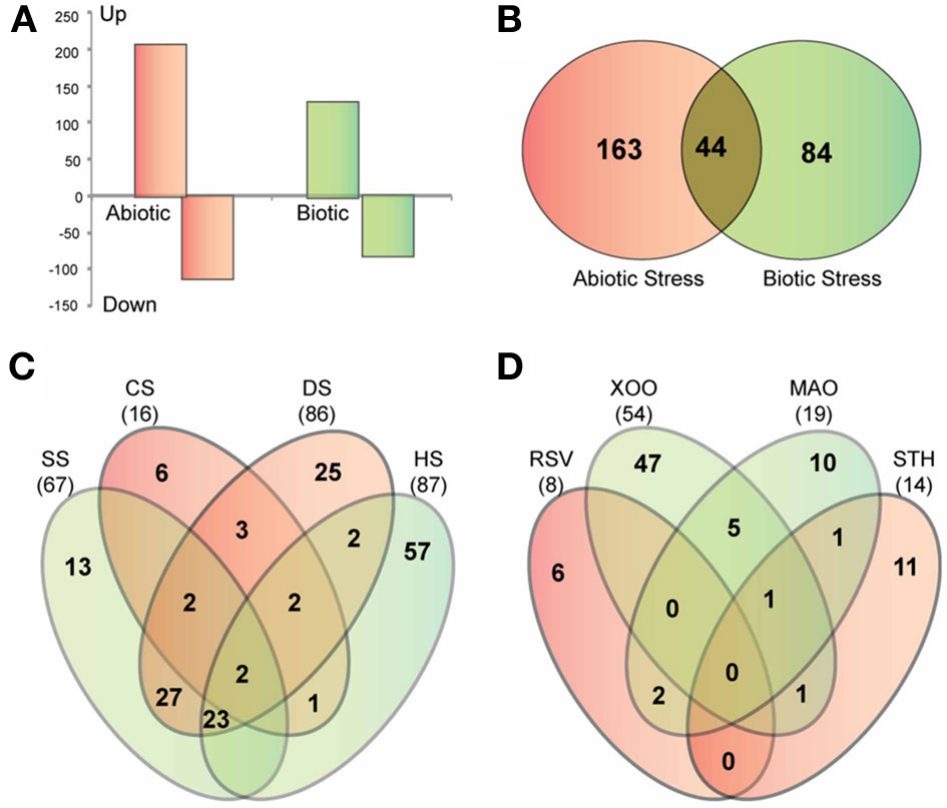
**Stress-responsive expression of glycoside hydrolase genes in rice. (A)** Graph showing the number of GH genes up-regulated and down-regulated in response to abiotic and biotic stresses. **(B)** Venn diagram showing the overlap between up-regulated genes in response to abiotic and biotic stresses. **(C,D)** Venn diagrams showing the overlap among four datasets of up-regulated genes in response to different biotic **(C)** and abiotic stress treatments **(D)**. SS, salt stress; CS, cold stress; DS, dehydration stress; HS, heat stress; *Xoo*, bacterial pathogen *Xanthomonas oryzae* pv. *oryzae*; *MAO*, fungal pathogen *Magnaporthe oryzae* strain Guy11; RSV, viral pathogen rice stripe virus; STH, parasitic plant *Striga hermonthica*.

To further elucidate the role of glycoside hydrolases in stress adaptation, we analyzed the expression of GH gene transcripts in response to four abiotic stress treatments, cold, heat, dehydration and salt, and four biotic stress treatments, bacterial infection caused by *Xanthomonas*, fungal infection caused by *Magnaporthe*, viral infection caused by rice stripe virus and a parasitic interaction with *Striga hermonthica*. The publicly available microarray-based expression data was downloaded from gene expression omnibus (GEO) repository of NCBI (http://www.ncbi.nlm.nih.gov/geo/). Differential expression analysis showed that 207 GH genes are up regulated, whereas, 112 are down regulated in response to at least one of the abiotic stresses (Figure 7A). Further dissection of up-regulated genes in response to individual stress treatments showed that a large number of GH family genes are induced in response to heat (87), dehydration (86) and salt (67) stress, while cold stress had the least impact on regulation of glycoside hydrolases (Figure 7C). Members of the GH17, GH18 and GH28 families are primarily up regulated in response to both salt and dehydration stress (Table S4). Although there is significant overlap among the up-regulated genes in response to heat, salt and dehydration stress, 57 genes were exclusively induced in response to heat stress. These genes include those encoding twelve GH1 family β-glucosidases, five GH9 family endoglucanases, five GH16 family xyloglucan endotransglycosylase hydrolases, seven GH17 family endo-β-1, 3-glucosidases and four GH18 family chitinases (Table S4). The GH1 family genes mainly implicated in metabolism of secondary metabolites have also been suggested to be involved in phytohormone activation (Minic, 2008). Ectopic expression of *AtBG1* that encodes β-glucosidase in *Arabidopsis* leads to accumulation of abscisic acid (ABA) and enhanced tolerance to abiotic stress (Lee et al., 2006). Recently, the rice GH1 family gene *Os9BGlu31* was shown to play role in stress adaptation by equilibrating the phenolpropenoid, flavonoid, and phytohormone glycoconjugates (Luang et al., 2013). Therefore, the observed significant induction of GH1 genes in response to heat stress might be associated with phytohormone signaling. In addition, the genes involved in triacylglycerol (GH5) and starch (GH13 and 14) degradation were notably up regulated in response to dehydration stress (Table S4).

In response to the biotic stresses analyzed, 128 GH genes were up regulated and 82 were down regulated (Figure 7A). Forty-four of the genes up regulated in response to biotic stresses were also induced in response to at least one of the abiotic stress treatments (Figure 7B). Most of these comprised of *Pathogenesis-related* (*PR*) genes of the GH17 family and genes encoding chitinases of the GH19 family. These genes present potential candidates to investigate overlap and crosstalk between stress-response signaling pathways in rice (Sharma et al., 2013).

Analysis of differential expression in response to stress treatments also revealed functional specializations of GH genes which otherwise appeared to be functionally redundant. For example, 11 tandemly duplicated genes of the GH18 family, clustered on chromosome 11, exhibited similar expression patterns during developmental stages (Figure 5A). However, only three of these genes were up regulated in response to abiotic stresses while another two were induced only in response to biotic stresses (Table S5). Therefore, environmental factors can, at least in some cases, be used to distinguish genes that appear functionally redundant under normal growth conditions.

Members of the GH17 family encoding endo-β-1, 3-glucosidases and chitinase-encoding genes of the GH18 and GH19 families were up regulated in response to fungal infection and parasitic interaction with *Striga*. The *PR* genes of the GH17 family, *PR2* genes, encode proteins that hydrolyze 1,3-β-D-glucosidic linkages in β-1, 3-glucans (Leubner-Metzger and Meins, 1999), whereas chitinases are known to hydrolyze β-1,4-N-acetyl-D-glucosamine linkages and contribute to plant defense systems by attacking the cell walls of insects, fungi and microbial pathogens (Collinge et al., 1993). The up-regulation of *PR2* genes and chitinases in response to these interactions indicate that degradation of cell wall components of pathogens is an important defense reaction in rice. Moreover, since fungal cell walls are generally comprised of both chitinases and β-1, 3-1,6-glucans, the combination of chitinases and β-1, 3-glucanases being expressed together suggest that they function together to degrade fungal cell walls (Minic, 2008). Degradation of pathogen cell walls also releases oligosaccharides which are recognized by plants and, in turn, activate plant defense responses (Minic, 2008).

Forty-seven genes were induced in response to *Xanthomonas* infection (Figure 7D) while the other three biotic interactions had much lesser impact on the expression of GH genes. Thirteen GH17 family genes were in particular, up regulated in response to *Xanthomonas* infection. A significant number of *PR* genes, eleven of the GH17 family and five genes encoding β-glucosidases of the GH1 family, were specifically up regulated in response to *Xanthomonas* infection. When we queried RiceNet with the genes up-regulated in response to infection with *Xanthomonas oryzae* pv. *oryzae*, (*Xoo)*, three of them, *Os01g71340, Os07g46280*, and *Os10g41550*, were found to be associated with several quality-control chaperones associated with endoplasmic reticulum including a heat shock protein 70 named binding immunoglobulin protein [BiP; (Park et al., 2010)], a ubiquitin ligase named XA21-binding protein 3 [ubiquitin ligase XB3; (Wang et al., 2006)], two DNaJ domain proteins, OsDjA4 and OsDjC79 (Sarkar et al., 2013), Calreticulin 3 (CRT-3) and *OsPDIL2-3* encoding protein disulfide isomerase. The evidence for their interaction with the queried genes comes from the mRNA co-expression of rice orthologs in *Arabidopsis* or literature-curated human protein interactions datasets in RiceNet (http://www.functionalnet.org/ricenet/RiceNet.v1.evidence_code.txt). Two of the proteins in the network predicted based on these associations, BiP and XB3, directly interact with XA21 (Wang et al., 2006; Park et al., 2010). XA21 is a rice receptor kinase that confers broad-spectrum resistance to *Xanthomonas oryzae* pv. *oryzae*. BiP and XB3 are required for XA21 protein stability and *XA21*-mediated resistance to *Xoo* (Wang et al., 2006; Park et al., 2010). Because N-glycosylation is important for correct folding, stability, and function of proteins that confer resistance to plant diseases, and it has been shown that XA21 is highly glycosylated (Park et al., 2010), the GH proteins in this network may be required for N-glycosylation of XA21 or other proteins involved in resistance to *Xoo*. Further experiments are required to decipher the precise role(s) of these genes and the proteins they encode.

The integration of transcriptomic data revealed the significant impact of environmental stresses on the regulation of cell wall-related genes in rice. Therefore, suggest that modulating the expression of cell wall-related genes will affect both saccharification yield and response to stress.

## Materials and methods

### Identification of rice GHs and database construction

The various enzymes involved in biosynthesis, modification and degradation of cell wall components across different taxonomic groups are catalogued in the frequently updated Carbohydrate-Active EnZymes (CAZy) database (Cantarel et al., 2009). All the members of the GH gene families of rice were downloaded from the CAZy database (http://www.cazy.org/Home.html). Additional members were identified in the Rice Genome Annotation Project database (RGAP, http://rice.plantbiology.msu.edu/) using a homology search and a Pfam (Finn et al., 2010) GH domain search with HMMER 2.3.2 using default parameters (Hughey and Krogh, 1996). The presence of a domain was confirmed in all the sequences using the Pfam (http://pfam.sanger.ac.uk/) and InterPro (http://www.ebi.ac.uk/interpro/) databases. The genes were classified into previously identified subfamilies of glycoside hydrolases (http://www.cazy.org/Glycoside-Hydrolases.html) based on sequence homology. Information about three-dimensional structures, clans, catalytic mechanisms, and enzyme activities for each family was procured from the CAZy database. The number of transcripts encoded by each family member was calculated based on the gene models predicted as part of the MSU RGAP (http://rice.plantbiology.msu.edu/index.shtml). The accession numbers of rice genes provided in MSU RGAP were used as gene names.

The design scheme for the Rice GH database is similar to those for the previously reported Rice kinase and GT databases (Dardick et al., 2007; Cao et al., 2008; Jung et al., 2010). The Rice GH database was constructed using the PHP programming language (http://php.net/) and MySQL database server (http://www.mysql.com/). The http address of the website for the Rice GH database is http://ricephylogenomics.ucdavis.edu/cellwalls/gh/. The curated data for the gene families, as well as various annotation information, are stored in a MySQL database system and can be accessed using SQL. The web platform is based on the Apache HTTP server (https://httpd.apache.org/) and its pages are generated using PHP (PHP: Hypertext Preprocessor) language (http://www.php.net/). Perl language and BioPerl modules (http://www.bioperl.org/wiki/Main_Page) were applied to manipulate data and convert different data formats. All of these procedures related to the rice GH database were executed on the Linux operating system. For each family with more than three members, full protein sequences were aligned using ClustalW version 2.0 (Larkin et al., 2007) with default options. Maximum likelihood trees were built using the default LG model (Le and Gascuel, 2008) in PhyML 3.0 (Guindon et al., 2010). Sequence information for each gene was downloaded from the Rice Genome Annotation project (http://rice.plantbiology.msu.edu/) and The Rice Annotation Project (http://rapdb.dna.affrc.go.jp/) websites. The NCBI BlastP search function of the rice GH database is linked to the protein blast option in NCBI (http://blast.ncbi.nlm.nih.gov/Blast.cgi). Information about sequence quality was obtained from the Rice Annotation Project Database (http://rice.plantbiology.msu.edu/). Orthologs of rice glycoside hydrolases were identified in eight dicot and four monocot genomes using Inparanoid version 4.1 (http://inparanoid51.sbc.su.se/cgi-bin/index.cgi). Information about transmembrane domains in the protein sequences was obtained from the TMHMM server version 2.0 (http://www.cbs.dtu.dk/services/TMHMM/). Plant-specific myristoylation predictor (http://plantsp.sdsc.edu/myrist.html) was used to identify the potential N-terminal myristoylation sites. The presence of N-terminal signal peptides was predicted using SignalP version 3.0 (http://www.cbs.dtu.dk/services/SignalP/). Chloroplast transit peptides were predicted using ChloroP version 1.1 (http://www.cbs.dtu.dk/services/ChloroP/). The subcellular localization of proteins was predicted using TargetP version 1.1 (http://www.cbs.dtu.dk/services/TargetP/). Information about the available transposon and T-DNA Insertional mutants in the National Institute of Agrobiological Sciences (NIAS) Tos17 Insertion Mutant Database (Miyao et al., 2003); the UCD Rice Transposon Flanking Sequence Tag Database with Ds Knockout (KO) lines (Kolesnik et al., 2004); the Oryza Tag Line (OTL) Database with Tos17 and T-DNA KO lines; the Rice Mutant Database (RMD) with T-DNA KO lines (Zhang et al., 2006); the Taiwan Rice Insertional Mutants Database (TRIM) with T-DNA KO lines; and the Postech Rice T-DNA Insertion Sequence Database with T-DNA KO and Activation (AC) tagging lines (An et al., 2003; Jeong et al., 2006) for all the members of the GH families was gathered from the OryGeneDB database (http://orygenesdb.cirad.fr/tools.html) and integrated with the rice GH database.

Gene expression information, comprised of digital northern data from available ESTs, MPSS tags and microarray platforms, was also integrated into the rice GH database. The MPSS data was downloaded from the Rice MPSS database [http://mpss.udel.edu/rice/; (Nakano et al., 2006)]; the values given represent transcripts per million (TPM) derived from the sum of 17 bp signatures. The raw microarray data, collected from a total of 105 rice experiments, was downloaded from the publicly accessible databases: Gene Expression Omnibus (GEO) at the National Center for Biotechnology Information [NCBI; (Barrett et al., 2011)], ArrayExpress at the European Bioinformatics Institute [EBI; (Parkinson et al., 2007)], Plant Expression Database [PLEXdb (Dash et al., 2012)] and also obtained from previously published experiments using NSF arrays (Jung et al., 2008). The data generated using Affymetrix arrays was normalized using the MAS5.0 algorithm contained in the R package (Ihaka and Gentleman, 1996). Two-channel data was normalized using Lowess (Locally weighted scatterplot smoothing). To normalize variation between arrays, MAD (median-absolute-deviation) scale normalization method contained in the R package (Wang et al., 2002) was used. The probe IDs from all the microarray platforms were mapped onto RAP (Rice Annotation Project; http://rapdb.dna.affrc.go.jp/) V3, RGAP (Rice Genome Annotation Project; http://rice.plantbiology.msu.edu/) V6 and KOME (Knowledge-based Oryza Molecular biological Encyclopedia) cDNAs using the BLAST tool of NCBI (http://blast.ncbi.nlm.nih.gov/Blast.cgi) as previously described (Cao et al., 2012). These expression data for all 437 rice GH genes have been integrated into the GH database.

### Identification of monocot- and rice-diverged GH encoding genes

Eight dicot [Arabidopsis (Arabidopsis thaliana), cucumber (Cucumis sativus), soybean (Glycine max), barrel clover (Medicago truncatula), monkey flower (Mimulus guttatus), poplar (Populus trichocarpa), castor bean (Ricinus communis) and grape (Vitis vinifera)] and four monocot [Brachypodium (Brachypodium distachyon), switchgrass (Panicum virgatum), Sorghum (Sorghum bicolor) and maize (Zea mays)] genomes were scanned using Inparanoid (http://inparanoid51.sbc.su.se/cgi-bin/index.cgi) for orthologs of rice genes. The sequence information from bacterial artificial chromosome (BAC) libraries, generated from the AP13 clone of switchgrass, was used for identifying GH orthologs in switchgrass (Sharma et al., 2012). The rice genes lacking corresponding orthologs in any of the eight dicot plant systems were considered monocot-diverged. The monocot-diverged glycoside hydrolase genes of rice lacking orthologs in any of the four monocot genomes analyzed were considered rice-diverged.

### Chromosomal distribution and duplication analysis

Chromosomal position coordinates, splicing variants and PASA status of genes was downloaded from RGAP V6 (http://rice.plantbiology.msu.edu/). Segmental paralogs of duplicated genes were identified using information available in RGAP release 4 (Ouyang et al., 2007). Tandemly duplicated genes were determined by calculating percent identities among GH genes lying in tandem. Glycoside hydrolase genes lying adjacent to each other or separated by only one gene and with >70% identity at the protein level were considered to be tandem duplicates. The percent homology between duplicated genes was determined using the EMBOSS Needle-Pairwise sequence alignment tool (http://www.ebi.ac.uk/Tools/psa/emboss_needle/). The chromosomal distribution of all the genes and segmental duplications was visualized using GenomePixelizer (Kozik et al., 2002). Expression data from duplicated genes were downloaded using the meta-analysis tool of the Rice Oligonucleotide Array Database (Cao et al., 2012) and imported into MeV microarray data analysis platform (http://www.tm4.org/mev/). The Pearson's correlation coefficient values among expression profiles of duplicated gene pairs were calculated using Microsoft excel. The hierarchical cluster analysis was performed in MeV (http://www.tm4.org/mev/) using the Euclidean distance metric and average linkage clustering method.

### Identification of genes preferentially expressed in vegetative or reproductive tissues and network analysis

Spatiotemporal expression data for all of the rice GH genes were downloaded from ROAD [http://www.ricearray.org/index.shtml;(Cao et al., 2012)]. The gene expression profiles were identified based on K-means clustering performed using MeV (http://www.tm4.org/mev/) and manual curation. The Hierarchical cluster diagram and heat maps were generated using MeV. The selected gene sets exhibiting developmental stage-preferential expression or those with high expression in developing tissues were queried in RiceNet (Lee et al., 2011) to identify the predicted interaction partners.

### Expression analysis in response to stress treatments

Data reflecting the expression of each GH gene in response to four abiotic stress treatments, salt (SS), cold (CS) and dehydration stress (DS; GSE6901) and heat stress (HS; GSE14275), as well as four biotic stress treatments, infection with a bacterial pathogen, *Xanthomonas oryzae* pv. *oryzae;* (*Xoo*; GSE16793), a fungal pathogen, *Magnaporthe oryzae* strain Guy11, (*MAO*; GSE18361), a viral pathogen, rice stripe virus (RSV; GSE12681) and interaction with the parasitic plant *Striga hermonthica*, (STH; GSE10373) were downloaded from the GEO (Gene Expression Omnibus) data repository of NCBI (http://www.ncbi.nlm.nih.gov/geo/). Each dataset in response to stress treatment was separately normalized using MAS 5.0 as described before and analyzed using Microsoft Excel to identify differentially expressed genes. For differential expression analysis, a cut off of = 2-fold differential expression at *P*-value = 0.01 was used.

### Conflict of interest statement

The authors declare that the research was conducted in the absence of any commercial or financial relationships that could be construed as a potential conflict of interest.

## Supplementary material

Figure S1 **Hierarchical display of spatiotemporal expression patterns of monocot-diverged glycoside hydrolase genes in rice**. The top row indicates the developmental stages/tissues analyzed as follows: PG, pre-germination seed; GS, germinating seedling; L1, 1st leaf; L2, 2nd leaf; L3, 3rd leaf; TI, Tillering initiation; T, Tillering stage; P1-P6, temporal stages of panicle development and S1-S5, temporal stages of seed development; GS, germinating seedling; PL, plumule; RA, radicle; SD, seedling; SH, shoot; L, leaf; S, stem; I, internode; R, root; SAM, shoot apical meristem; FL, flag leaf; SP, spikelet; PL, palea/lemma; ST, stigma; OV, ovary; AN, anther; ES, embryo sac; E, embryo; EN, endosperm; DS, dry seed. The locus IDs are given on the left and gene family names are given on the right of the heat map. The information about rice-diverged genes is given on the right of the heat map. The color legend is given at the base where green signifies very low-level expression, black indicates medium-level expression, and red represents high-level expression. The numbers in the color legend correspond to relative log_2_ expression values.

Table S1 **List of monocot and rice-diverged glycoside hydrolase genes of rice**.

Table S2 **List of tandemly arrayed glycoside hydrolase genes of rice**.

Table S3 **List of segmental duplicated glycoside hydrolase genes of rice**.

Table S4 **List of glycoside hydrolase genes up regulated in response to abiotic stresses**.

Table S5 **List of glycoside hydrolase genes up regulated in response to biotic stresses**.
