# Supplementary figures and images for: Construction of a rice glycoside hydrolase phylogenomic database and identification of targets for biofuel research
Source: Front Plant Sci. 2013 Aug 26;4:330. doi: 10.3389/fpls.2013.00330 (PMC3752443; doi:10.3389/fpls.2013.00330)

## Slide 1
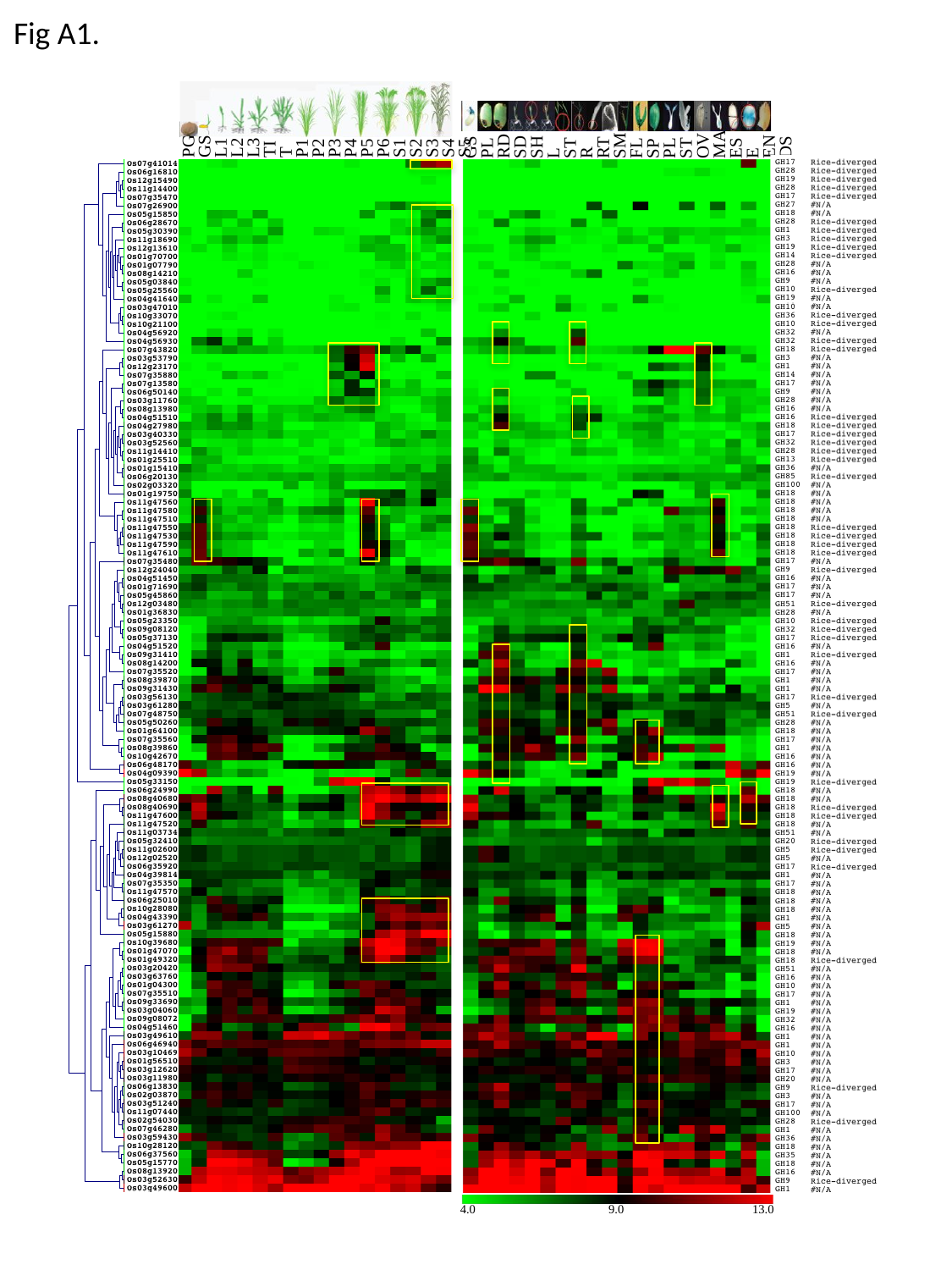

Fig A1.
PG
GS
L1
L2
L3
TI
T
P1
P2
P3
P4
P5
P6
S1
S2
S3
S4
S5
GS
PL
RD
SD
SH
L
ST
R
RT
SM
FL
SP
PL
ST
OV
MA
ES
E
EN
DS
4.0
9.0
13.0

Supplement: Figure S1 — Hierarchical display of spatiotemporal expression patterns of monocot-diverged glycoside hydrolase genes in rice. The top row indicates the developmental stages/tissues analyzed as follows: PG, pre-germination seed; GS, germinating seedling; L1, 1st leaf; L2, 2nd leaf; L3, 3rd leaf; TI, Tillering initiation; T, Tillering stage; P1-P6, temporal stages of panicle development and S1-S5, temporal stages of seed development; GS, germinating seedling; PL, plumule; RA, radicle; SD, seedling; SH, shoot; L, leaf; S, stem; I, internode; R, root; SAM, shoot apical meristem; FL, flag leaf; SP, spikelet; PL, palea/lemma; ST, stigma; OV, ovary; AN, anther; ES, embryo sac; E, embryo; EN, endosperm; DS, dry seed. The locus IDs are given on the left and gene family names are given on the right of the heat map. The information about rice-diverged genes is given on the right of the heat map. The color legend is given at the base where green signifies very low-level expression, black indicates medium-level expression, and red represents high-level expression. The numbers in the color legend correspond to relative log2 expression values. [file Presentation1.PPTX]
